# Pathological granuloma fibrosis induced by agar-embedded *Mycobacterium abscessus* in C57BL/6JNarl mice

**DOI:** 10.3389/fimmu.2023.1277745

**Published:** 2023-12-11

**Authors:** Shiu-Ju Yang, Chih-Hao Hsu, Chi-Yun Lai, Pei-Chu Tsai, Yung-Deng Song, Chang-Ching Yeh, Yih-Yuan Chen, Horng-Yunn Dou

**Affiliations:** ^1^ National Institute of Infectious Diseases and Vaccinology, National Health Research Institutes, Zhunan, Miaoli, Taiwan; ^2^ Pathology Core Laboratory, National Health Research Institutes, Zhunan, Miaoli, Taiwan; ^3^ Department of Biochemical Science and Technology, National Chiayi University, Chia-Yi, Taiwan

**Keywords:** pulmonary granuloma diseases, fibrosis, mycobacterium abscessus, hypoxia, immunocompetent mice

## Abstract

**Introduction:**

Pulmonary granuloma diseases caused by *Mycobacterium abscessus* (*M. abscessus*) have increased in past decades, and drug-resistance in this pathogen is a growing public health concern. Therefore, an animal model of chronic granuloma disease is urgently needed.

**Methods:**

In this study, *M. abscessus* embedded within agar beads (agar-AB) was used to develop such a model in C57BL/6JNarl mice.

**Results:**

Chronic infection was sustained for at least 3 months after agar-AB infection, visible granulomas spread in the lungs, and giant cells and foamy cells appeared in the granulomas. More importantly, pulmonary fibrosis progressed for 3 months, and collagen fibers were detected by Masson trichrome staining. Further, inducible nitric oxide synthase (iNOS) was highly expressed within the alveolar space, and the fibrosis-mediator transforming growth factor beta (TGF-β) began to be expressed at 1 month. Hypoxia-inducible factor (HIF-1α) expression also increased, which aided in normalizing oxygen partial pressure.

**Discussion:**

Although the transient fibrosis persisted for only 3 months, and the pulmonary structure resolved when the pathogen was cleard, this pulmonary fibrosis model for *M. abscessus* infection will provide a novel test platform for development of new drugs, regimens, and therapies.

## 1 Introduction

Granulomatous lung diseases are a group of heterogeneous disorders histologically characterized by compact aggregates of histiocytes (macrophages) in the lungs, and often containing neutrophils, lymphocytes, plasma cells, foamy cells, and multinucleated giant cells (1, 2). They can be classified as noninfectious or infectious. Noninfectious causes include sarcoidosis, Crohn’s disease, berylliosis, and granulomatosis with polyangiitis, among others (1). Most infectious pulmonary granuloma cases are caused by mycobacteria, which includes *M. tuberculosis* and non-tuberculous mycobacteria (NTM) (1). Unlike *M. tuberculosis*, NTM are highly abundant in the environment, such as in soil and water, and are usually non-pathogenic to humans and animals. However, NTM can affect individuals who are elderly, have a genetic pulmonary disease (such as cystic fibrosis), are immunodeficient, or have preexisting lung conditions (such as chronic obstructive pulmonary disease or bronchiectasis) (3, 4). In recent decades, NTM infections have been increasing worldwide, especially in developed countries (5). Based on a prospective study in Taiwan on mycobacterial prevalence during 2010-2014, the most sensitive age group is 65 years and older (6). Further analysis of the data revealed *M. abscessus* and *M. avium* to be the main NTM strains in Taiwan (6). Despite having many available antibiotics, treatment failure remains high. Moreover, *M. abscessus* prevalence is contributing to a rise of drug resistance (7, 8), thus limiting treatment choices. Therefore, an animal model of NTM infection is urgently needed.

*M. abscessus* is an emerging pathogen and a growing health concern among both immunocompetent and immunocompromised patient populations. Several *M. abscessus*-infection animal models have been developed for different targets, such as mycobacteria-susceptible C3HeB/FeJ mice, immunocompromised SCID mice, nude mice, interferon-γ knockout (KO) mice, and GM-CSF KO mice (9–12), including usage of corticosteroids to increase the susceptibility of mice (13), and a zebrafish model (14). All of these models have improved our knowledge of *M. abscessus* pathology; however, an immunocompetent mouse model is still needed to extend the range of available models.

In this study, we used the method of agar-embedded mycobacteria following previously published protocols (15, 16) from the laboratories of Dr. I. Kukavica-Ibrulj and Dr. C. Riva to successfully establish an NTM infection model in C57BL/6JNarl mice. The agar beads provide a protective shell and release bacteria slowly to cause chronic infection mimicking the disease process in humans. Interestingly, the mice developed transient pulmonary fibrosis at 2-3 months post infection, which we did not expect to see. This new animal model could provide a platform for anti-fibrotic drug and regimen development.

## 2 Materials and methods

### 2.1 Bacterial cultures and agar beads-embedded mycobacteria

**Figure 1:**
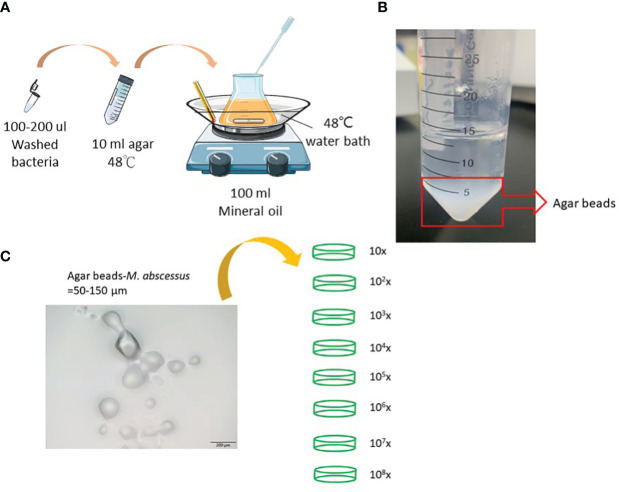
Preparation of agar-embedded *M. abscessus* (agar-AB). **(A)** Preparation of agar-AB was modified from references (15, 16). **(B)** Agar-AB suspended in PBS. **(C)** Morphology of agar-AB by light microscopy. Scale bar = 200 μm. The bacterial concentration of agar-AB beads was determined by dilution (10^1^ to 10^8^-fold) and plating.

*Mycobacterium abscessus* strain ATCC 19977 was used in this study. Cells were maintained on Middlebrook 7H9 medium (Difco Laboratories, Detroit, MI, USA) supplemented with 0.5% glycerol, 0.05% Tween 80, and 10% albumin-dextrose- catalase or on solid Middlebrook 7H11 medium (Difco Laboratories) supplemented with oleic acid-albumin-dextrose-catalase. Agar-embedded *M. abscessus* (agar-AB) was prepared according to published protocols (15, 16). Briefly, log-phase *M. abscessus* cells (CFU = 10^8-9^) were washed with phosphate-buffered saline (PBS) and resuspended in 200 μl of PBS. Bacterial suspension, mineral oil, and 1.5% TSA-agar were incubated in a water bath at 48°C with stirring at medium speed for 5 min (**Figure 1A**). Then the mixture was moved quickly to a 4°C cold-room and stirring was continued for 20 min, and then transferred to ice for another 20 min. After three washes with PBS, the agar beads were stored at 4°C for 1 week (**Figure 1B**).

### 2.2 Mice and agar-AB infection

Female C57BL/6JNarl and C3H/HeNCrNarl mice aged 6 to 8 weeks were purchased from the National Laboratory Animal Center (Taipei, Taiwan). All mice were kept in individually ventilated cage environments at the Animal Center of the National Health Research Institutes (Maoli, Taiwan). Animal experiments were reviewed and approved by the National Health Research Institutes Institutional Animal Care and Use Committee (NHRI-IACUC). We conducted experiments according to guidelines set out by the Association for Assessment and Accreditation of Laboratory Animal Care International (AAALAC). First, mice were challenged with 10-10^8^ CFU of *M. abscessus* or 10^3-5^ CFU of agar-AB per mouse at week 0 by intratracheal inoculation to find the optimal condition. Subsequently, mice were challenged with 10^4^ CFU of agar-AB per mouse. After agar-AB infection, murine blood and lung were harvested for pathological and immunological assays at months 1, 2, 3, 4, 5, 6, and 7.

### 2.3 Bacterial culture

Half of each lung tissue sample was minced and passed through a 70-μm mesh to produce single-cell suspensions in 5 ml of saline (cell survival rate >99.5% by hemocytometer). CFU values were determined by using 100 μl of cell mixture dilution on 7H10 agar plates. Each plate contained 100 μl of tissue homogenate, and each sample was titrated for three dilutions (10× and 100×) performed in triplicate. Plates were kept at 37°C for 3 to 7 days. The colony number was counted and presented as a value per lung/mouse.

### 2.4 Acid-fast stain

Samples of lung tissue were fixed in formalin and embedded in paraffin using routine methods. The sections were then deparaffined with UltraClear (J.T. Baker, Belgium), and then stained with carbolfuchsin-acid alcohol solution (BASO Histological Acid Fast Stain, Taiwan).

### 2.5 Hematoxylin and eosin stain, masson trichrome stain, and immunohistochemical staining

Samples of lung tissue were fixed in formalin and embedded in paraffin using routine methods, and the sections were then stained with H&E and Masson Trichrome stain. Tissue was processed by the core pathology facility at the National Health Research Institutes (Maoli, Taiwan). For each time point, lung tissue from three to six animals was processing in the core facility. Fibrosis grade was scored by using the modified scale (grade 0-8) (17). For IHC, paraffin sections were rehydrated and retrieved with 100 mM citrate buffer (pH 6.0) at 100°C for 5 min or Tris/EDTA buffer (pH 9.0) at 100°C for 20 min. After blocking the peroxidase activity and background (IHC/ICC kit, BioVision, Milpitas, CA, USA), serial sections were incubated with primary antibody anti-iNOS (RRID: AB_301857, Abcam, ab15323, Cambridge, UK), anti-TGF-β (RRID: AB_2811115, Proteintech, 21898-1-AP, Rosemount, IL, USA), and anti-HIF-1α (Novus Biologicals, NB100-105, Centennial, CO, USA), and stained following the manufacturer’s protocol (BioVision). Finally, the sections were colored with the chromogen DAB and counterstained with hematoxylin.

### 2.6 Hypoxia detection

Before mouse sacrifice, the mice were weighed and intraperitoneally injected with 10 mM EF5 compound, which was prepared by following the protocol of the EF5 Hypoxia Detection kit, Alexa Fluor^®^ 488 (Millipore, CA, USA). Samples of lung tissue were fixed in 4% paraformaldehyde and embedded in paraffin using routine methods, and the sections were then stained with anti-EF5 antibody (RRID: AB_2756853, clone ELK3-51 Alexa Fluor^®^ 488 conjugate, Millipore, CA, USA). The detection procedure was as described in (18). An oxygen partial pressure (pO_2_) standard curve was produced by incubating tissue in 0% O_2_ (Baker Ruskinn, Concept Plus anaerobic station, UK) and 20% O_2_ for 1 hour, and then following the same histochemistry treatment as above. The pixels of green fluorescence generated by the 0% and 20% O_2_-incubated tissue sections were then counted. The counts were obtained from 3 sections stained with EF-5 per sample and at least 6-10 random views per section. The experimental pO_2_ values were calculated by using an internal difference method of the standard curve.

### 2.7 Statistical analysis

All results are presented as mean ± SEM for three or six mice per group. The statistical significance between the experimental groups was assessed using a two-way analysis of variance (ANOVA) with a Bonferroni *post hoc* test. The differences were considered significant for *P* values less than 0.05. Statistical tests were performed using GraphPad Prism version 6.0 (GraphPad Software, La Jolla, CA, USA).

## 3 Results

### 3.1 Preparation and titration of agar bead-embedded *M. abscessus*

To generate a more effective chronic infection in mice, *M. abscessus* strain ATCC 19977 cells were embedded in 1.5% agarose gel (agar-AB) according to published protocols (see Materials and Methods, **Figure 1**) and titrated for bacterial concentration. The size of the agar-AB beads was about 50-150 µm, as determined by light microscopy (**Figure 1C**). The agar beads were prepared fresh for each usage.

### 3.2 Mild cell infiltration in C57BL/6JNarl mice post *M. abscessus* ATCC 19977 infection

To determine the optimal infection dose of *M. abscessus* ATCC 19977 strain in C57BL/6JNarl mice, 10-10^8^ CFU of *M. abscessus* per mouse were inoculated intratracheally and the mice were sacrificed at week 4 (**Supplementary Figure 1A**). All mice survived until week 4 except those in the 10^8^ CFU group, which died at day 4 and day 5 (**Supplementary Figure 1A**), indicating that 10^8^ CFU is a lethal dose for C57BL/6JNarl mice. The CFU/lung of the surviving mice for the 10^5^, 10^6^, and 10^7^ doses were 1.667 ± 0.333, 222.333 ± 111.667, 1555.333 ± 1059.729, respectively (**Supplementary Figure 1B**). The CFU numbers for the other infection doses (0-10^4^) were all zero (**Supplementary Figure 1B**). These results show that lung colonization by *M. abscessus* does not succeed in immunocompetent C57BL/6JNarl mice. In addition, the severity of cell infiltration, as revealed by H&E staining, increased in a dose-dependent manner for 10^4^ to 10^7^ CFU, compared with the control (0 CFU) (**Supplementary Figure 1C**). However, even up to 10^7^ CFU the inflammation still was mild (**Supplementary Figure 1C**).

### 3.3 Optimal dose for agar-AB infection in C57BL/6JNarl and C3H/HeNCrNarl mice

**Figure 2:**
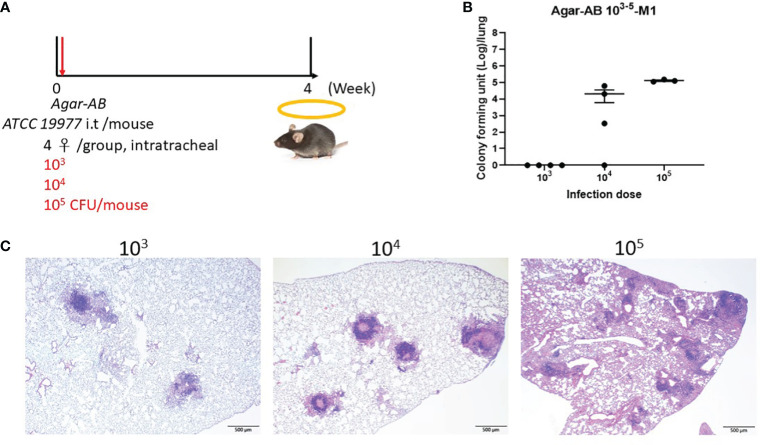
Determination of the optimal dose of agar-AB for establishment of granuloma disease in C57BL/6JNarl mice. C57BL/6JNarl mice were infected with 10^3^-10^5^ CFU of agar-AB intratracheally at week 0 (n = 4). After infection, at week 4, the mice were sacrificed. **(A)** Schematic diagram of the agar-AB infection protocol. **(B)** Colony forming units (CFU) of bacterial growth in mouse lung for each tested dose. **(C)** Histological examination of granuloma presence. Lung samples were fixed in 3.7% formaldehyde, embedded in paraffin, sectioned, and stained with H&E. Scale bar = 500 μm.

Next, to determine the optimal infection condition for agar-AB, doses of 10^3^, 10^4^, and 10^5^ CFU per mouse were inoculated intratracheally into C57BL/6JNarl and C3H/HeNCrNarl mice at week 0, and the mice were sacrificed at week 4 (**Figure 2A**). In C57BL/6JNarl mice, the CFU/lung values for the 10^3^, 10^4^, and 10^5^ infection doses were 0 ± 0, 2.066×10^4^ ± 1.457×10^4^, 1.3011×10^5^ ± 1.209×10^4^, respectively (**Figure 2B**). Compared with the non-agar embedded *M. abscessus* (**Supplementary Figure 1B**), the CFU values were 10- to 100-fold increased. In H&E-stained lung sections, the 10^4^ and 10^5^ groups showed remarkable granuloma structures compared with the groups inoculated with unembedded bacteria (**Figure 2C**). We decided to use an infection dose of 10^4^ CFU per mouse for further study because this dose produced ‘perfect’ granuloma structures. Empty beads were not detected in control C57BL/6JNarl mice; however, they were seen clearly in the bronchi of control C3H/HeNCrNarl mice (mouse 1 and 3, green arrows, **Supplementary Figure 2**) at 1 month post infection, although their presence did not cause cell infiltration. Some beads were also obvious in lung tissue of C3H/HeNCrNarl mice that received the 10^6^ dose (mouse 3, green arrow, **Supplementary Figure 2**). We assumed that rare cell infiltration may have occurred in C57BL/6JNarl mouse lung after inoculation with empty beads; however, we found no evidence for this. As a precaution, we applied the same treatment condition to C3H/HeNCrNarl mice, but found no cell infiltration and some empty beads in the H&E sections. The C3H/HeNCrNarl strain is another immunocompetent mouse control for inoculation with empty beads.

### 3.4 Persistent chronic granuloma in C57BL/6JNarl mice post agar-AB infection

**Figure 3:**
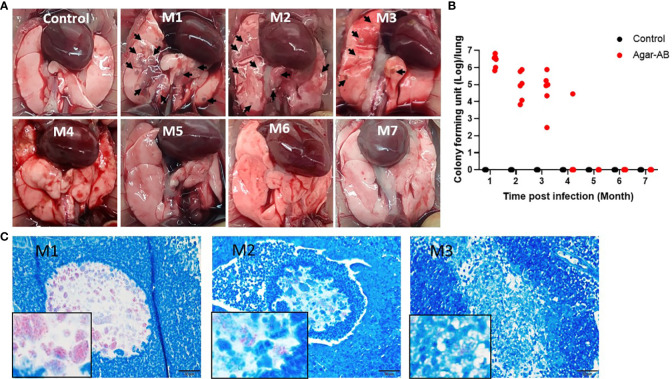
Persistent chronic granuloma in C57BL/6JNarl mice post agar-AB infection. C57BL/6JNarl mice were infected with 10^4^ CFU of agar-AB intratracheally at week 0 (n = 3 for control, and n = 6 for agar-AB). After infection at months 1, 2, 3, 4, 5, 6, and 7 (M1-M7), the mice were sacrificed. **(A)** Gross anatomy of lungs of normal control and post infection mice at M1 to M7. Granulomas appeared at M1-M3 (black arrows). **(B)** Colony forming units (CFU) of bacterial growth in mouse lung at M1 to M7. **(C)** Acid-fast staining was performed at M1, M2, and M3. The red color reveals mycobacteria in lung tissue counterstained with hematoxylin (blue). The boxes show enlarged views. Scale bar = 20 μm.

We next tested agarose-embedded *M. abscessus* for its efficacy to induce long-term chronic infection in mice. At 1 week post infection, the agar-AB-infected mice occasionally showed shortness of breath, but returned to normal breathing. Mice were sacrificed at months 1, 2, 3, 4, 5, 6, and 7 (M1-M7) post agar-AB infection. At M1, the gross anatomy showed numerous clear or gray granulomas in the lung, compared with normal control mice (empty beads, **Figure 3A**). At M2 and M3, a few granulomas were observed on the lung surface, but the granuloma number was decreased (**Figure 3A**). At M4, M5, M6, and M7, the granuloma had disappeared, and the lung structure was almost back to normal and pink in color (**Figure 3A**). The CFU counts were very high in the previous three months (M1-M3), 3.17×10^6^ ± 8.965×10^5^, 2.619×10^5^ ± 1.335×10^5^, and 1.898×10^5^ ± 1.168×10^5^, respectively (**Figure 3B**). At M4, the CFU number had decreased to 4.723×10^3^ ± 4.722×10^3^ (**Figure 3B**). However, at M5, M6 and M7, the CFU counts were all 0 ± 0 (**Figure 3B**). Finally, we used an acid-fast stain (Ziehl Neelsen stain) to evaluate *M. abscessus* in agar-AB beads localized in lung bronchi. At M1, we observed red-stained mycobacteria present in agar beads, surrounded by inflammatory cells. At M2, red mycobacteria still were present in the beads; however, the beads were beginning to be destroyed by infiltrating cells. At M3, red mycobacteria were spread into the inflammation area, and the agar beads no longer existed (**Figure 3C**). Thus, agar-AB infection sustained stable chronic inflammation for up to 3 months, and the bacterial load persisted at about 10^6^-10^5^ CFU at M1-M3.

**Figure 4:**
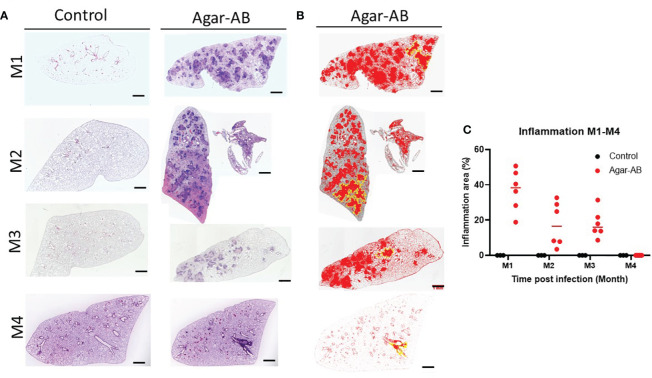
Mild to severe inflammation in C57BL/6JNarl mice post agar-AB infection. C57BL/6JNarl mice were infected with 10^4^ CFU of agar-AB intratracheally at week 0 (n = 3 for control, and n = 6 for agar-AB). After infection at months 1, 2, 3, and 4 (M1-4), the mice were sacrificed. **(A)** H&E staining reveals multiple granuloma lesions in agar-AB-infected lung, compared with their absence in control mice. Scale bar = 1 mm. **(B)** Graphic of ImageJ analysis showing the inflamed areas in mouse lungs. The red regions correspond to the inflammation. The yellow outlines indicate the borders of the inflamed areas. H&E sections were converted to 8-bit grayscale images, and then adjusted to the threshold. The yellow lines surrounding the red areas were delineated by hand. **(C)** The inflammation area (%) was calculated by ImageJ software.

All H&E stained sections were scanned using a light microscope (**Figure 4A**) and the inflammation areas were calculated by ImageJ software (**Figures 4B, C**). All control-group mice at M1-M3 presented 0% inflammation. In agar-AB-infected mice, at M1 the inflammation area was about 20-50%, at M2 the inflammation area was slightly down (5-40%), and at M3 it was the same as M2 (5-40%). The overall H&E staining results were similar to those in **Figures 2**.

### 3.5 Granuloma formation, giant cells, foamy cells, and fibrosis in agar-AB infected mice

**Figure 5:**
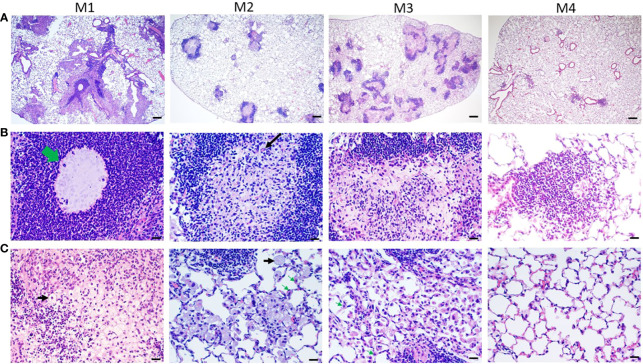
Pathological granuloma lesions in C57BL/6JNarl mice post agar-AB infection. C57BL/6JNarl mice were infected with 10^4^ CFU of agar-AB intratracheally at week 0 (n = 3 for control, and n = 6 for agar-AB). At months 1, 2, 3, and 4 (M1-4) after infection, the mice were sacrificed. **(A)** H&E staining reveals multiple granuloma lesions in agar-AB-infected lung post infection at M1, 2, 3, and 4. Scale bar = 200 µm. **(B)** High magnification (400×) images of granuloma showing neutrophil infiltration surrounding the bacterial agar beads (green arrow) at M1, multinucleated giant cells (black arrow) at M2, and fibroblasts surrounding dead cell debris in granuloma lesions at M3. Scale bar = 20 µm. **(C)** High-magnification (400×) images of circumambient granuloma showing small cellular debris and cubic alveolar cells at M1, foamy cells (black arrows) and vacuous enlarged alveolar cells (green arrows) at M2 and M3, and back-to-normal alveolus at M4. Scale bar = 20 µm.

Histological characterization of agar-AB-infected lungs was performed post infection for M1-M7. Agar beads were located in bronchial lumens, and severe inflammation began by day 7 (which is the first time point of sacrifice, data not shown) and persisted for almost 4 months (**Figure 5A**), as did granuloma formation (**Figure 5**). At M1, agar-AB beads were undigested (green arrow, **Figure 5B**) and located in the parenchyma around bronchi, and some vessels were surrounded by neutrophils, and infiltration by macrophages, T and B cells was evident. Enlarged and cuboid type I and II alveolar cells filled the pulmonary septum, space, and bronchi, some of them damaged (evident as cellular debris) and destined to be phagocytized by immune cells (**Figure 5C**). At M2, granuloma structures were matured, giant cells appeared within the granuloma (black arrow, **Figure 5B**), and spindle fibroblasts were circulating in the center. Some vacuous alveolar macrophages were spread outside of the granulomas, and alveolar cells had regenerated and were back to normal (**Figure 5C**). At M3, more spindle fibroblasts replaced some of the cell debris in the center core and surrounding area, and some debris of phagocytosis by neutrophils-phagocytes was present inside as well (**Figure 5B**), and vacuolated histiocytes were located at the parenchyma and alveolar space (**Figure 5C**). At M4, the masses of cell infiltrates had almost disappeared and only traces of inflammation remained around the bronchi, and the alveolar septum and structure were almost back to normal (**Figures 5A-C**).

### 3.6 Collagen fibers expressed in pathological granuloma

**Figure 6:**
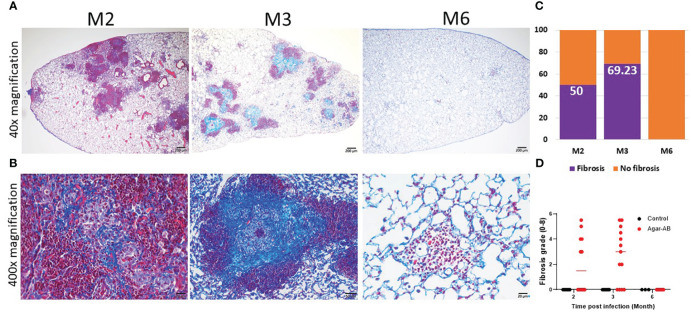
Collagen fibers in granuloma lesions in C57BL/6JNarl mice post agar-AB infection at months 2, 3 and 6 (M2, M3, M6). C57BL/6JNarl mice were infected with 10^4^ CFU of agar-AB intratracheally at week 0 (n = 3-6 for control, and n = 6-13 for agar-AB). After infection at M2, M3, and M6, the mice were sacrificed and Masson Trichrome staining was performed on lung sections. **(A)** Lung sections showing granulomas and infiltration by cells surrounding the lesions. Scale bar = 200 µm. **(B)** High-magnification (400×) images showing blue fibers inside and fibroblasts mixing with dead-cell debris in the granuloma lesions, and red infiltrating cells surrounding the lesions. Scale bar = 20 µm. **(C)** Fibrosis percentage in mice post agar-AB infection at M2, M3, and M6. **(D)** Fibrosis grade in mice post agar-AB infection at M2, M3, and M6.

From H&E staining, we observed spindle cells and pink, non-nuclear areas in granulomas, which underwent fibrosis in lesions at M2 and M3; therefore, we performed Masson Trichrome staining of lung sections at M2, M3, and M6. The collagen fibers (stained blue) of the septum were three times thicker than normal (17), and the cells were stained red. At M2 at 40× magnification, fibrosis was not apparent (**Figure 6A**); however, at 400×, mild fibrosis could be seen (**Figure 6B**). At M3, bold and colorful blue staining was present, full in the core of the granuloma (**Figure 6B**). But at M6, the bold blue color was no longer seen (**Figures 6A, B**), and the tissue had returned to normal pulmonary septum thickness with some infiltration by cellular debris (**Figure 6B**). The fibrosis rate is shown in **Figure 6C**, calculated as the number of fibrosis-positive mice/total number of mice in each group (M2, M3, M6). For example, at M2, 6 of 12 mice had fibrosis, for a fibrosis rate of 50%. At M3, 9 of 13 mice had fibrosis, 69.23%. Following the pulmonary fibrosis grade definitions from 2008 (17), we scored the severity of fibrosis at M2, M3, and M6. Most of the fibrosis mice had scores of 4-5 (**Figure 6D**). Mild fibrosis started to proceed at M2, and mature fibrosis appeared at M3, at which time the structure of the pulmonary septum began to return to normal. At M6, excess collagen fibers were no longer apparent, and the structure of the pulmonary alveoli was back to normal.

### 3.7 Level of iNOS, TGF-β, and HIF-1α expressed in granuloma

**Figure 7:**
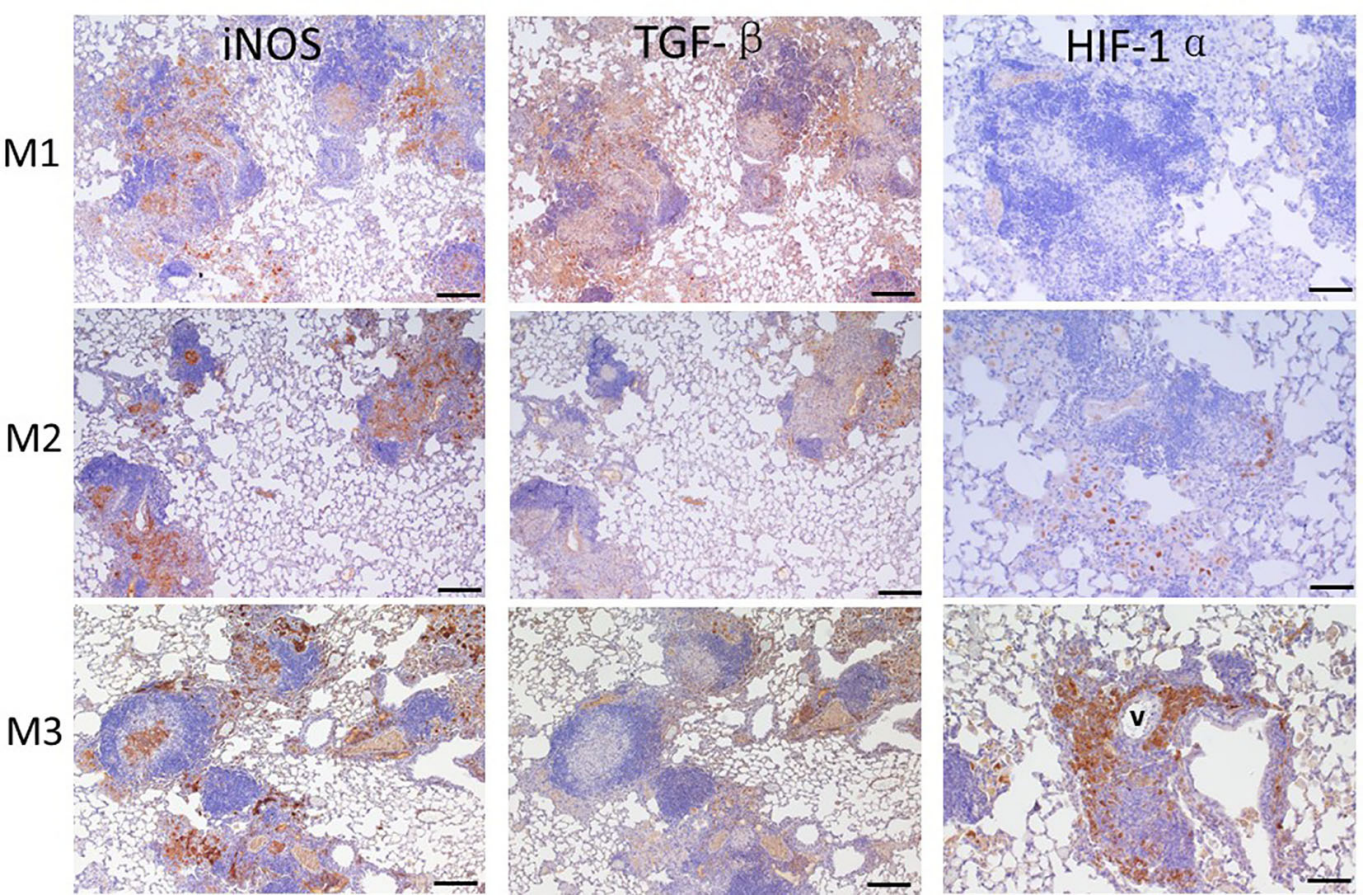
Expression of iNOS, TGF-β, and HIF-1α in granuloma lesions in C57BL/6JNarl mice post agar-AB infection at months 1, 2, and 3 (M1-3). C57BL/6JNarl mice were infected with 10^4^ CFU of agar-AB intratracheally at week 0 (n = 3 for control, and n = 6 for agar-AB) and sacrificed at M1, M2 and M3, and IHC staining was performed. The sections were stained with primary antibodies (iNOS, TGF-β, and HIF-1α), and then colored with the chromogen DAB and counterstained with hematoxylin. V, vessel; scale bar = 100 µm.

After agar-AB infection, a robust cell inflammatory response, which included T cells, B cells, neutrophils, and macrophages, was evident in the pulmonary alveolar space, bronchi, and septum (data not shown). Inducible nitric oxide synthase (iNOS), which is expressed by type I macrophages and mediates Th1 and Th17 immune responses, was also detected. iNOS was highly expressed at M1 (early stage of infection) and throughout M2 and M3 (**Figure 7**). Therefore, *M. abscessus* infection induces strong Th1 and Th17 immunity.

**Figure 8:**
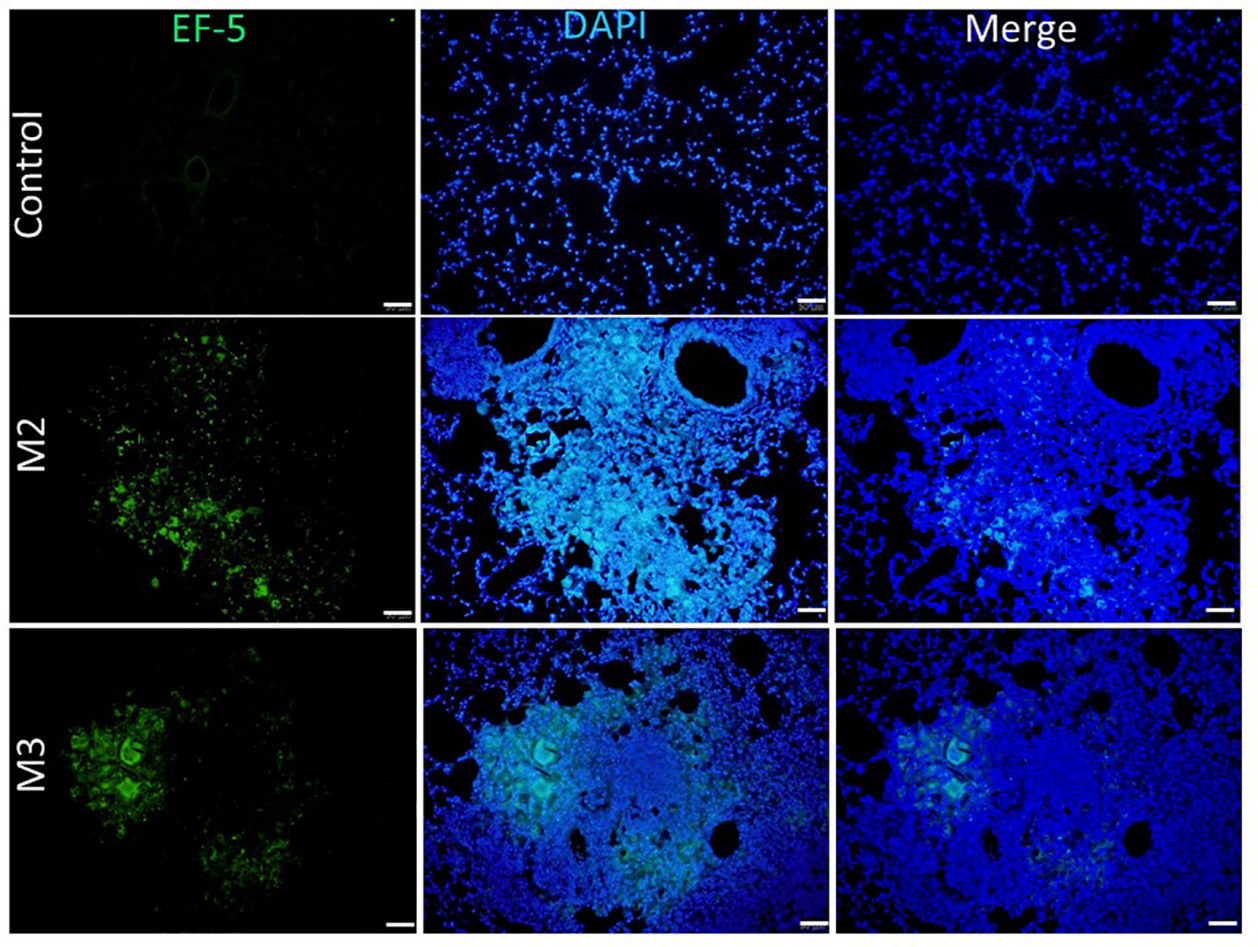
Expression of hypoxia in granuloma lesions in C57BL/6JNarl mice post agar-AB infection at month 2 and 3 (M2 and M3). C57BL/6JNarl mice were infected with 10^4^ CFU of agar-AB intratracheally at week 0 (n = 3 for control, and n = 6 for agar-AB). After infection, at M2 and M3, the mice were sacrificed and EF5 staining was performed. The sections were stained with primary antibodies (ELK antibody conjugated Alexa Fluor^®^ 488). Scale bar = 50 μm.

TGF-β is a key factor involved in mediating fibrogenesis through smad7 and β-catenin (19), and also restricts T-cell expansion within tuberculous granuloma (20). At M1, we detected high TGF-β expression in the infiltrated alveolar space and surrounding the granuloma (**Figure 8**). However, TGF-β expression was decreased at M2 and M3, especially outside the core granuloma (**Figure 7**). In addition, iNOS and TGF-β showed opposite expression patterns at M2 and M3 (**Figure 7**). In other words, TGF-β was induced at an early stage of infection and maintained a low level of expression in the chronic period.

Based on the significant fibrosis, as seen in **Figure 6**, we expected hypoxia to be present in fibrotic regions of the lungs. Hypoxia-induced factor-1α (HIF-1α) is a regulator of oxygen homeostasis and responds to falling oxygen levels in tumors and fibrotic tissue (21–24). At M1, HIF-1α expression was very low, present in the alveolar space sporadically. At M2, its expression was increased in areas surrounding granulomas. At M3, HIF-1α was highly expressed, involving almost half the granuloma and surrounding the vessel (**Figure 7**). This means that HIF-1α expression is highly correlated to fibrosis severity (**Figures 6A, B**) following infection and disease progression.

### 3.8 Hypoxia induced by agar-AB expressed in the granuloma

To detect and measure the oxygen supply under hypoxia *in vivo*, we used an EF5 hypoxia detection method (18). Briefly, upon injection into animal tissues, EF5 selectively binds to hypoxic cells and forms adducts. A mouse monoclonal antibody, clone ELK3-51, which is directly conjugated to Alexa Fluor^®^ 488, is then used to selectively bind the EF5 adducts. Control mice that were intratracheally inoculated with empty beads showed a normal pulmonary structure and aerobic condition [pressure of O_2_ (pO_2_) is 20%]: the fluorescence signal of EF-5 binding was extremely low (black by visualization, with the raw pixel values of the image ranging from 0.002-0.186) in the control lung (**Figure 8**). In contrast, mice that were intratracheally inoculated with Agar-AB showed green fluorescence signals in the alveolar space and bronchi, as well as some within the granuloma at M2 and M3 (**Figure 8**).

## 4 Discussion

Here we describe the first pulmonary fibrosis model for *M. abscessus* infection. The method we used, embedding bacteria within agar beads, was refined from the original studies of Kukavica-Ibrulj et al. and Riva et al. for *P. aeruginosa* (15) and *M. abscessus* (16), respectively. The agar bead size is critical, and our beads were around 50-150 μm in diameter, similar in size to those used by Riva et al. During several rounds of testing, we removed any large and unmixed agar from around the edge of Erlenmeyer flask, if present. Furthermore, the optimal infection dose 10^4^ CFU per mouse may be a key factor for fibrosis development, as shown by histopathological data (**Figure 2C**). In general, small bacterial inocula would be cleared easily, and high doses may induce severe inflammation and even cause mouse death. The process of evolving from a granuloma structure to fibrosis formation requires slow release of bacteria from a suitably sized bead and an optimal concentration of the pathogen.

In this study, fibrosis occurred and was maintained for 3 months following chronic *M. abscessus* infection (**Figures 6A, B**). The pathology was initiated at pathogen infection, in the context of tissue injury and repair as the immune system fought back. After pathogen clearance, the pulmonary alveolar structure recovered to normal. Several factors are required for the fibrosis process, a type of wound-healing in which connective tissue replaces normal parenchymal tissue: severe inflammation to cause tissue damage and repair over and over again, and expression of the pro-fibrotic mediator TGF-β and other drivers such as platelet-derived growth factor and IL-10 (25–27). In our study, iNOS was highly expressed at the beginning of infection at M1, and continued to be expressed at M2 and M3, indicating that iNOS-expressing macrophages within the granuloma were kept activated to kill the pathogen. The bacterial load was about 10^7^ CFU/ml at M1, then decreased to about 10^4-5^ CFU/ml at M2 and M3, and dropped again to about 10^3^ CFU/ml at M4 (**Figure 3B**). It is likely that such active macrophages cleared all of the foreign threat, as observed post infection at M4 to M7. In addition, TGF-β was expressed at an early stage post infection (at M1), but fell to a lower level at M2 and M3. However, even the low level of TGF-β was sufficient to induce the proliferation and activation of fibroblasts, which deposited extracellular matrix into the surrounding connective tissue at M2 and M3 (**Figure 7**). This increased the fibrosis rate from 50% at M2 to 66.7% at M3 (**Figure 6C**) as well as the fibrosis grade (**Figure 6D**).

Although caseous necrosis centers within granulomas did not appear in this model, which usually are obvious in tuberculosis (TB) and NTM pulmonary infection, dense collagen accumulation and hypoxia did occur in these experimental granulomas, and the former may cause the latter. First, HIF-1α expression was rare at M1, increased at M2, and was high at M3 (**Figure 7**), which correlates to the progression of fibrosis stage (**Figure 6A**). Interestingly, when we measured the pulmonary pO2 in mouse tissue by EF-5 staining, lesion hypoxia was lessened at M3 compared to M2. However, green EF-5 fluorescent signal still was observed at M2 and M3 (**Figure 8**). The whole fibrosis process in this model is completely reversible while the bacteria were cleared by the immune system.

Establishment of *M. abscessus* lung infection in a mouse model is problematic. We demonstrated that *M. abscessus* can persist in the lungs of mice, specifically immunocompetent mice. Furthermore, we demonstrated not only a long-term chronic mycobacterial infection for 3 months, but also a transient fibrosis model. Limitations include that the model showed no caseous necrosis as seen in TB patients, and some typical abnormal pulmonary structures were not evident in this model; moreover, the transient fibrotic tissue resolved to a normal structure at 4 months post infection. However, this is the first fibrosis model caused by a Risk Group 2 (RG2) mycobacterial species that could be handled easily in an Animal Biosafety Level 2 laboratory, thereby saving resources and labor compared to pathology studies in an Animal Biosafety Level 3 laboratory. Our model could also be used as a platform for preclinical testing of new drugs and treatment regimes.

## Data availability statement

The original contributions presented in the study are included in the article/**Supplementary Material**. Further inquiries can be directed to the corresponding author.

## Ethics statement

The animal study was approved by National Health Research Institutes Institutional Animal Care and Use Committee, NHRI-IACUC-110042-A. The study was conducted in accordance with the local legislation and institutional requirements.

## Author contributions

SY: Conceptualization, Data curation, Formal analysis, Methodology, Project administration, Validation, Visualization, Writing – original draft. CH: Data curation, Methodology, Writing – original draft. CL: Data curation, Methodology, Validation, Writing – original draft. PT: Data curation, Methodology, Writing – original draft. YS: Data curation, Methodology, Writing – original draft. CY: Data curation, Methodology, Writing – original draft. YC: Data curation, Methodology, Writing – review & editing. HD: Conceptualization, Supervision, Validation, Visualization, Writing – review & editing.
