# Supplementary material for: Pathological granuloma fibrosis induced by agar-embedded Mycobacterium abscessus in C57BL/6JNarl mice
Source: Front Immunol. 2023 Dec 11;14:1277745. doi: 10.3389/fimmu.2023.1277745 (PMC10749312; doi:10.3389/fimmu.2023.1277745)
Supplement: Supplementary file 1 [file DataSheet_1.docx]

Supplementary Material

# Supplementary Data

## Bacterial cultures and agar beads-embedded *Mycobacteria*

*Mycobacterium abscessus* strain ATCC 19977 was used in this study. Cells were maintained on Middlebrook 7H9 medium (Difco Laboratories, Detroit, MI, USA) supplemented with 0.5% glycerol, 0.05% Tween 80, and 10% albumin-dextrose- catalase or on solid Middlebrook 7H11 medium (Difco Laboratories) supplemented with oleic acid-albumin-dextrose-catalase. Agar-embedded *M. abscessus* (agar-AB) was prepared according to published protocols [14], [15]. Briefly, log-phase *M. abscessus* cells (CFU = 10^8-9^) were washed with phosphate-buffered saline (PBS) and resuspended in 200 μl of PBS. Bacterial suspension, mineral oil, and 1.5% TSA-agar were incubated in a water bath at 48℃ with stirring at medium speed for 5 min (Figure 1A). Then the mixture was moved quickly to a 4℃ cold-room and stirring was continued for 20 min, and then transferred to ice for another 20 min. After three washes with PBS, the agar beads were stored at 4℃ for 1 week (Figure 1B).

## Mice and agar-AB infection

Female C57BL/6JNarl and C3H/HeNCrNarl mice aged 6 to 8 weeks were purchased from the National Laboratory Animal Center (Taipei, Taiwan). All mice were kept in individually ventilated cage environments at the Animal Center of the National Health Research Institutes (Maoli, Taiwan). Animal experiments were reviewed and approved by the National Health Research Institutes Institutional Animal Care and Use Committee (NHRI-IACUC). We conducted experiments according to guidelines set out by the Association for Assessment and Accreditation of Laboratory Animal Care International (AAALAC). First, mice were challenged with 10-10^8^ CFU of *M. abscessus* or 10^4-6^ CFU of agar-AB per mouse at week 0 by intratracheal inoculation to find the optimal condition. Subsequently, mice were challenged with 10^4^ CFU of agar-AB per mouse. After agar-AB infection, murine blood and lung were harvested for pathological and immunological assays at months 1, 2, 3, 4, 5, 6, and 7.

## Bacterial culture

Half of each lung tissue sample was minced and passed through a 70-μm mesh to produce single-cell suspensions in 5 ml of saline (cell survival rate >99.5% by hemocytometer). CFU values were determined by using 100 μl of cell mixture dilution on 7H10 agar plates. Each plate contained 100 μl of tissue homogenate, and each sample was titrated for three dilutions (10× and 100×) performed in triplicate. Plates were kept at 37°C for 3 to 7 days. The colony number was counted and presented as a value per lung/mouse.

# Supplementary Figures and Tables

For more information on Supplementary Material and for details on the different file types accepted, please see [here](https://www.frontiersin.org/guidelines/author-guidelines#supplementary-material).

## Supplementary Figures


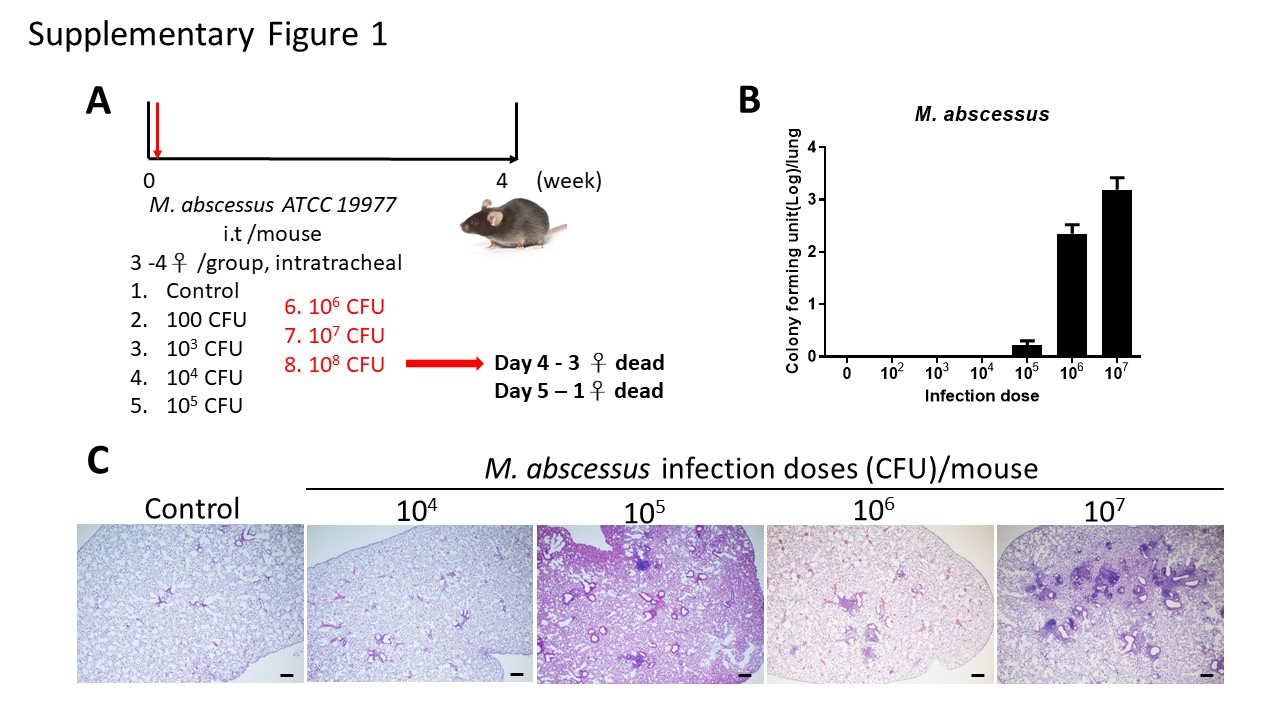


**Supplementary Figure 1.** Test of reference strain *M. abscessus* ATCC 19977 infection in C57BL/6JNarl mice. C57BL/6JNarl mice were infected with different doses of *M. abscessus* 19977 intratracheally at week 0 (100-10^8^ CFU/mouse, n = 3 to 4). After infection, at week 4 the mice were sacrificed. (A) Schematic diagram of the infection protocol. (B) Colony forming units (CFU) of bacterial growth in mouse lung. (C) Histological examination. Lung samples were fixed in 3.7% formaldehyde, embedded in paraffin, sectioned, and stained with H&E. Scale bar = 200 μm.


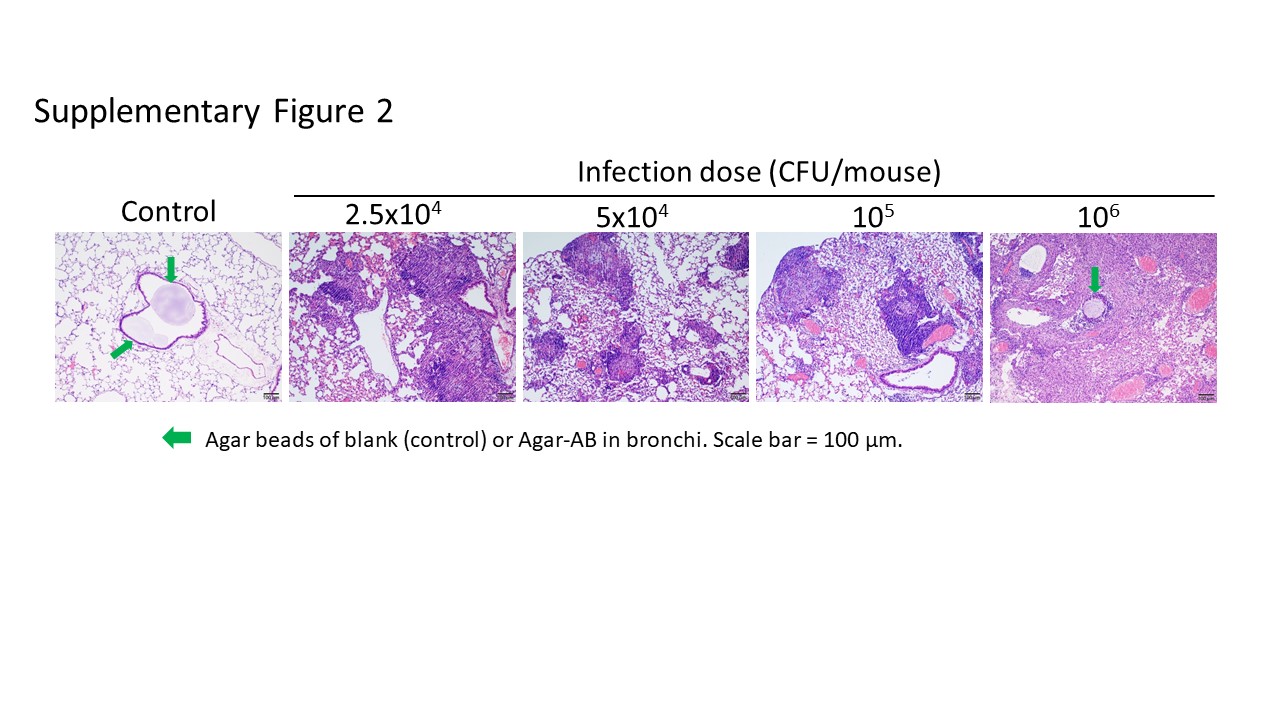


**Supplementary Figure** **2.** Morphology of different challenge doses of agar-AB in C3H/HeNCrNarl mice. C3H/HeNCrNarl mice were infected with 2.5×10^5^ to 10^6^ CFU of agar-AB intratracheally at week 0 (n = 3 for control, and n = 3 for agar-AB). Lung samples were fixed in 3.7% formaldehyde, embedded in paraffin, sectioned, and stained with H&E post infection at month 1; Green arrows: agar beads of blank (control) or Agar-AB in bronchi. Scale bar = 100 μm.
